# Myositis, Ganglioneuritis, and Myocarditis with Distinct Perifascicular Muscle Atrophy in a 2-Year-Old Male Boxer

**DOI:** 10.3389/fvets.2018.00020

**Published:** 2018-02-20

**Authors:** Paul M. Rossman, Stephanie A. Thomovsky, Ryan M. Schafbuch, Ling T. Guo, G. D. Shelton

**Affiliations:** ^1^Department of Veterinary Clinical Sciences, Purdue University College of Veterinary Medicine, West Lafayette, IN, United States; ^2^Indiana Animal Disease Diagnostic Laboratory, West Lafayette, IN, United States; ^3^Comparative Neuromuscular Laboratory, Department of Pathology, School of Medicine, University of California, San Diego, La Jolla, CA, United States

**Keywords:** megaesophagus, myositis, myocarditis, endocarditis, ganglioneuritis

## Abstract

A 2-year-old male, intact Boxer was referred for chronic diarrhea, hyporexia, labored breathing, weakness and elevated creatine kinase, and alanine aminotransferase activities. Initial examination and diagnostics revealed a peripheral nervous system neurolocalization, atrial premature complexes, and generalized megaesophagus. Progressive worsening of the dog’s condition was noted after 36 h; the dog developed aspiration pneumonia, was febrile and oxygen dependent. The owners elected humane euthanasia. Immediately postmortem biopsies of the left cranial tibial and triceps muscles and the left peroneal nerve were obtained. Postmortem histology revealed concurrent myositis, myocarditis, endocarditis, and ganglioneuritis. Mixed mononuclear cell infiltrations and a distinct perifascicular pattern of muscle fiber atrophy was present in both muscles. This is a novel case of diffuse inflammatory myopathy with a distinct perifascicular pattern of atrophy in addition to endocarditis, myocarditis, and epicarditis.

## Background

Inflammatory myopathies are relatively common in dogs and may be localized to specific muscle as in masticatory myositis, extraocular myositis, and glossitis, or be a generalized disorder including immune-mediated polymyositis (PM), dermatomyositis, infectious myositis, and as a paraneoplastic syndrome (1). Certain breeds may be predisposed to PM including the Viszla (2), Newfoundland (3), and Boxer (3). A few case reports have described the concurrent presentation of PM with myocarditis (4), with tongue muscle and masticatory muscle inflammation (5), and with gastrointestinal muscle inflammation (2). Here, we describe an unusual case of PM in a Boxer with concurrent megaesophagus, myocarditis, endocarditis, and ganglioneuritis. A distinct pattern of perifascicular atrophy was found in the skeletal muscles. Perifascicular atrophy is the occurrence of small muscle fibers at the periphery of a fascicle that are atrophic with some fibers regenerating, and are thought to reflect ischemic changes secondary to disease of the vessels (6). This pattern is found in most, but not all, cases of human dermatomyositis (6) but not in familial canine dermatomyositis (7).

## Case Presentation

Written informed consent was obtained from the owners of the dog featured in this case report. A 2-year-old male, intact Boxer was presented to the local veterinarian for a 1-day history of diarrhea and hyporexia. Fecal flotation and Giardia ELISA tests were negative. Treatment was initiated with oral metronidazole (10 mg/kg PO every 12 h) and probiotics. No improvement was reported following 4 days of treatment. Repeat physical examination revealed bilateral ptosis of the lower eyelids along with bilateral miotic pupils. A complete blood count, chemistry panel, and urinalysis were submitted and showed increases in alanine aminotransferase (ALT, 239 IU/L, reference 12–118), aspartate aminotransferase (AST, 369, reference 15–66), and a marked increase in creatine kinase activity (CK, 11,823 IU/L reference 50–895). Testing for infectious diseases including blastomyces antigen by enzyme immunoassay and tick-related diseases by polymerase chain reaction (PCR) were negative. Clindamycin (5.3 mg/kg PO every 12 h), minocycline (7 mg/kg PO every 12 h), and topical NeoPolyDex ointment (OS every 12 h) were prescribed. Twenty-four hours later, the dog developed labored breathing, weakness, and a cardiac arrhythmia was noted. The dog was referred to Purdue University Small Animal Hospital for further evaluation.

Physical examination at the time of referral revealed tetraparesis and an intermittent cardiac arrhythmia. The dog was bright and alert with a decreased body condition score (3/9), normothermic (rectal temperature 102.5°F) and had a normal heart rate (118°bpm), respiratory rate (24 breaths/min), and character. Cardiac auscultation revealed an intermittent arrhythmia that on electrocardiogram was atrial premature contractions. The arrhythmia was not consistently detected on repeat examinations. Thoracic auscultation was unremarkable but a cough was occasionally noted. Neurologic examination revealed ambulatory tetraparesis with no obvious ataxia. Conscious proprioception was normal in all four limbs. Hopping was normal during the initiation phase, but slow on all four limbs during the follow-through phase consistent with weakness. The remainder of the neurologic examination including cranial nerves was normal. Neurolocalization was to the neuromuscular system. A myopathy was considered more likely than a neuropathy or disorder of neuromuscular transmission as reflexes were normal and the CK activity was persistently elevated.

**Figure 1:**
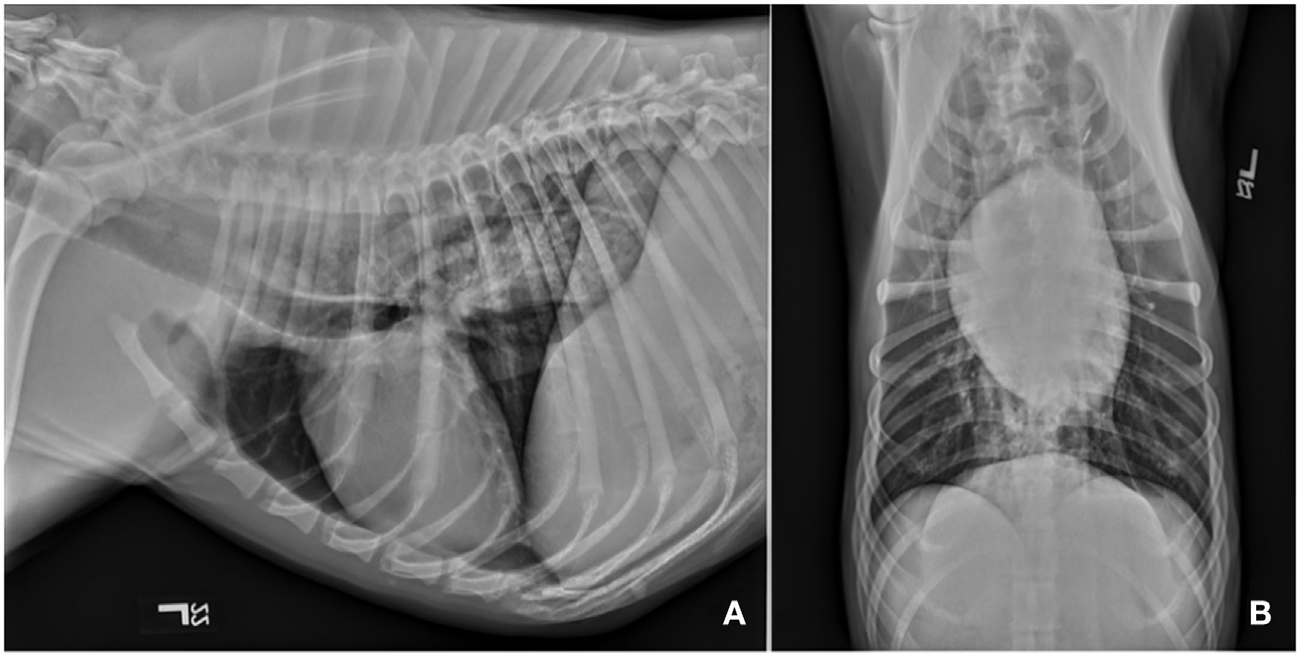
Survey thoracic radiographs including left lateral **(A)** and ventrodorsal **(B)** views revealed diffuse dilation of the cervical and thoracic portions of the esophagus. There are no obvious abnormalities associated with the pulmonary parenchyma, cranial mediastinum or vasculature, or the cardiac silhouette. Diffuse megaesophagus was confirmed.

Repeat CBC, biochemistry panel, and serum CK activities were performed. The ALT and CK activities were further increased from initial values; 365 IU/L (reference 3–69) and 19,242 IU/L (22–491), respectively. Baseline electrocardiogram revealed atrial premature complexes. Thoracic radiographs showed generalized megaesophagus with no abnormalities noted in the pleural space, cranial mediastinum, or pulmonary parenchyma (Figure 1). Abdominal ultrasound showed small hyperechoic splenic nodules along with medial iliac and jejunal lymphadenomegaly. Aspirates of the spleen and medial iliac lymph nodes were performed. Cytology was consistent with lymphoid hyperplasia.

The dog was hospitalized and treated with intravenous fluids, cerenia (1 mg/kg IV every 24 h) and amoxicillin/clavulanic acid (22 mg/kg IV every 8 h). With the history of regurgitation and radiographic evidence of megaesophagus, frequent small meals were given in a Bailey chair.

Thirty-six hours following referral, the dog became acutely febrile (104.1°F). An alveolar lung pattern with air bronchograms in the right middle, right caudal, and left cranial lung lobes was observed on thoracic radiographs in addition to evidence of a pneumothorax. The pneumothorax was suspected to be a result of pulmonary necrosis secondary to the aspiration pneumonia. This is considered a rare cause of pneumothorax. Enrofloxacin (5 mg/kg IV every 12 h) was initiated. Over the next 12 h, the dog became progressively weaker, oxygenation deteriorated, and bilateral nasal cannulas were placed for oxygen supplementation. The dog developed bilateral epistaxis, moderate to marked increased inspiratory effort, and bradypnea (12 breaths/min). Arterial blood gas was performed and showed a partial pressure of oxygen of 39 mmHg (90–100 mmHg) and partial pressure of carbon dioxide to be 32 mmHg (35–45 mmHg). Clotting times (PT/PTT) were within normal limits.

Electrophysiologic testing including electromyogram, measurement of motor nerve conduction velocity, and repetitive nerve stimulation were recommended, followed by peripheral nerve and muscle biopsies. Due to the aspiration pneumonia and rapidly declining respiratory status, the owners declined further testing and humane euthanasia was performed. Immediately postmortem, samples of the left cranial tibial and triceps muscles, and left peroneal nerve, were collected. The muscles were chilled and shipped under refrigeration to the Comparative Neuromuscular Laboratory or immersion fixed into neutral buffered formalin. The peroneal nerve was immersion fixed in neutral buffered formalin. A complete necropsy was performed at Purdue University.

### Muscle and Nerve Histopathology and Immunofluorescent Staining

**Figure 2:**
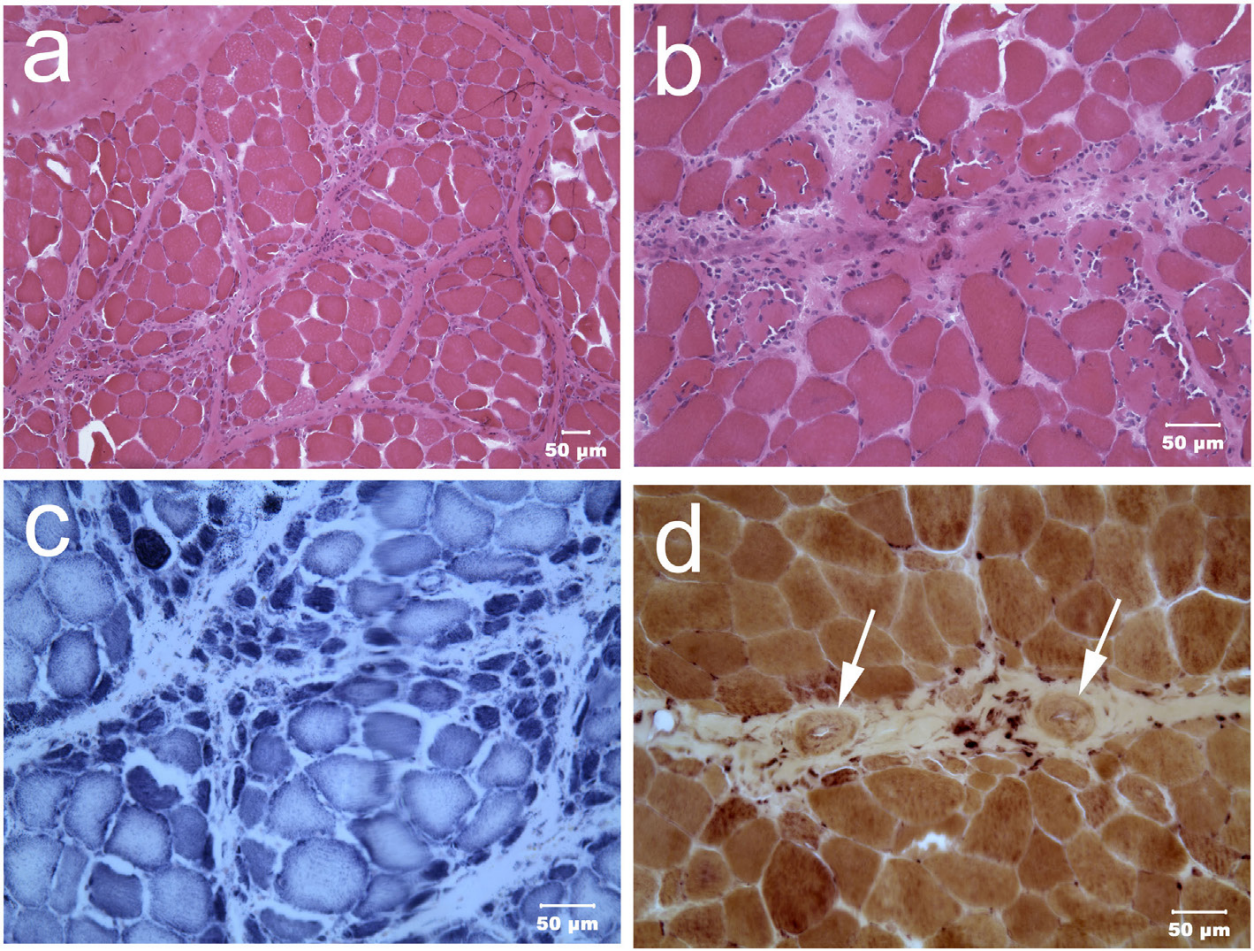
Cryosections from the triceps muscle at low [H&E stain **(A)**] and higher [H&E stain **(B)**] power illustrate the marked variability in myofiber size with atrophic fibers having a distinct perifascicular distribution. Scattered necrotic fibers undergoing phagocytosis are also evident. Many perifascicular fibers show intense dark blue staining with the oxidative reaction NADH-TR **(C)**. Small caliber blood vessels [**(D)** esterase reaction] with thickened walls (dark arrows) were present within the perimysium. A cluster of brown stained esterase reactive macrophages were adjacent to one of the vessels.

Immediately upon receipt, chilled muscles were flash frozen and cryosections evaluated with a standard panel of histochemical stains and reactions (6). A moderate variability in myofiber size was present in both muscles with atrophic fibers having a round shape and a marked perifascicular distribution (Figure 2). Most atrophic fibers were of histochemical type 2 C using the ATPase reaction for fiber typing. Small intramuscular nerve branches were normal in appearance. Multifocal areas of mixed mononuclear cell infiltration (lymphocytes and acid phosphatase and esterase reactive macrophages) were present having an endomysial and perimysial distribution. Eosinophils were not observed. Scattered necrotic myofibers were undergoing phagocytosis. No abnormalities were identified in the peroneal nerve. A diagnosis was made of moderately severe inflammatory myopathy/myositis with a distinct perifascicular pattern of atrophy.

**Figure 3:**
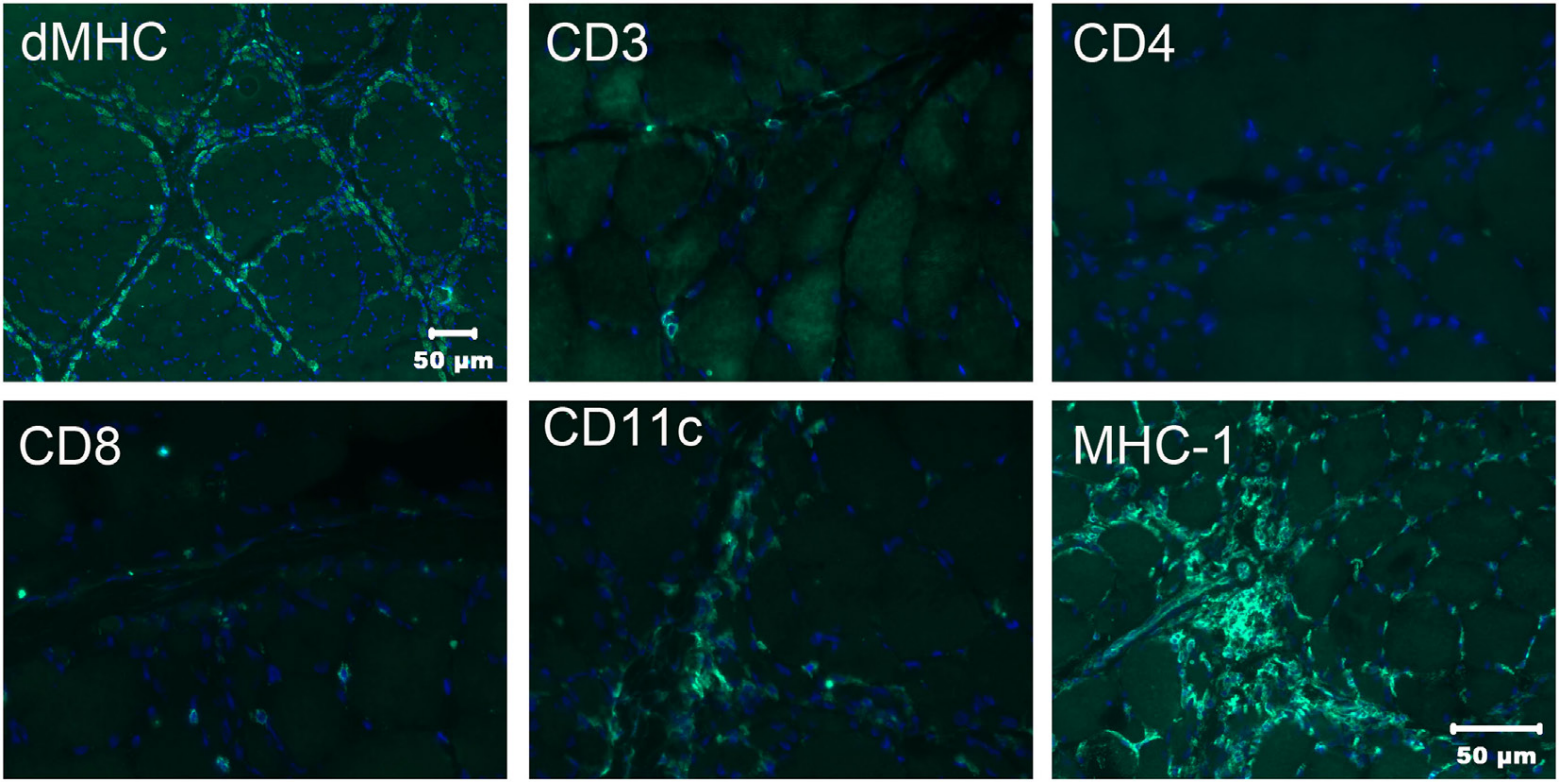
Immunofluorescence staining of frozen sections from the triceps muscle using a monoclonal antibody against developmental myosin heavy chain (dMHC) highlights the perifascicular distribution of immature muscle fibers. Scattered cells were highlighted with the antibodies against CD3 and CD8 leukocyte antigens but no CD4 positive cells were noted. Numerous cells were highlighted with the CD11c antibody against macrophages and dendritic cells. Using the antibody against MHC I, muscle sarcolemmal and internal labeling of myofibers was evident in some but not all myofibers. Bar in MHC I image also = 50 µm for CD3, CD4, CD8, and CD11c images.

To better characterize the population of infiltrating cells and confirm the nature of the atrophic fibers having a perifascicular distribution, immunofluorescent staining was performed using monoclonal antibodies against canine leukocyte antigens (8) and developmental myosin heavy chain (dMHC) (Developmental Studies Hybridoma Bank, University of Iowa, Figure 3) The perifascicular pattern of muscle fiber atrophy was highlighted with the antibody against dMHC, confirming the immaturity of the atrophic fibers (Figure 3). Scattered CD3 and CD8 positive T cells were present. Numerous CD11c positive cells were highlighted and MHC I staining was prominent.

### Necropsy Results

**Figure 4:**
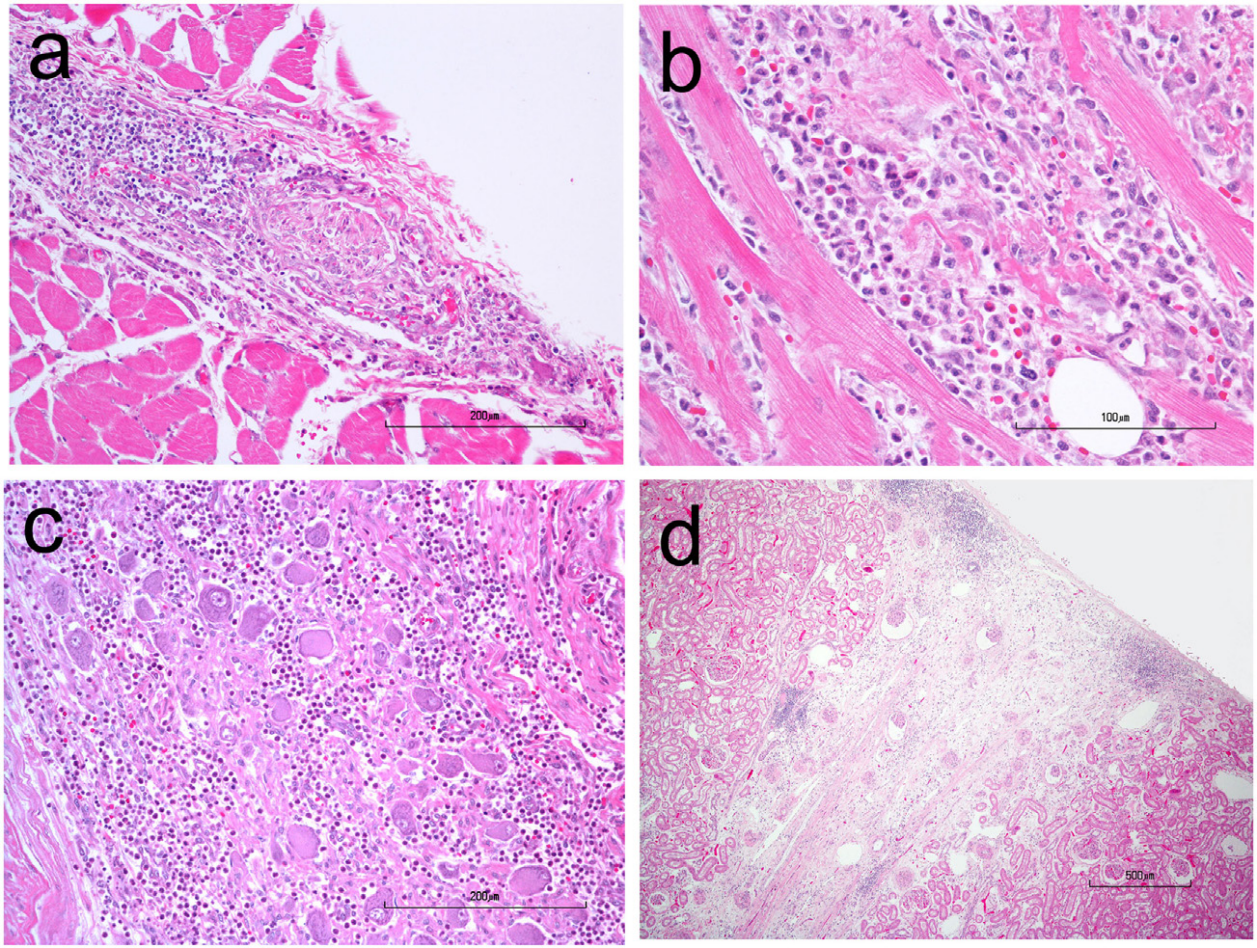
H&E stained photomicrographs showing infiltration and partial replacement of the esophageal myenteric plexus **(A)**, heart **(B)**, and vagus ganglion **(C)** by a population of neutrophils, macrophages, lymphocytes, and eosinophils. Also notice the peripheralization of Nissl substance and central chromatolysis of some neurons in the vagus ganglion. **(D)** A chronic, wedge-shaped infarct is present within the renal cortex.

**Figure 5:**
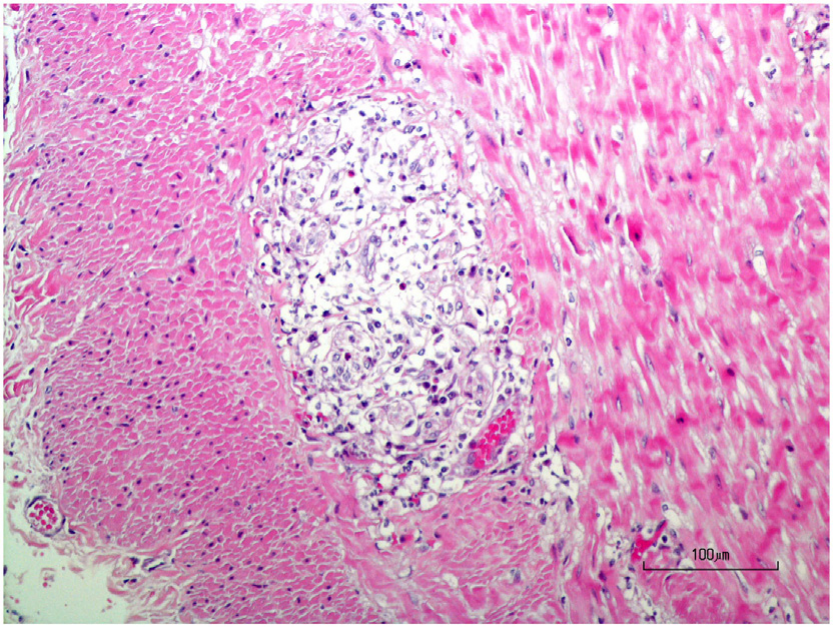
H&E stained photomicrograph showing infiltration and partial replacement of the colonic myenteric plexus by a population of neutrophils, macrophages, lymphocytes, and eosinophils.

A necropsy was performed following euthanasia. Muscles examined included: appendicular skeletal muscle (triceps and quadriceps muscle), tongue, diaphragm while nerve tissue examined included: nerve plexuses (Meissner’s/submucosal and Auerbach’s muscularis propria), sciatic, peroneal, ulnar, various ganglia. Necropsy revealed myositis, endocarditis, myocarditis and epicarditis, and ganglioneuritis within the plexuses of the colon, esophagus, and vagus nerve (Figure 4). The myositis was pleocellular with an increased population of eosinophils. Due to the infiltration of scattered eosinophils in various soft tissues, sections of frozen heart, and skeletal muscle were tested for *Neospora caninum* using PCR testing and were negative. A perifascicular distribution of atrophy was observed in muscle tissue in the more acutely affected fascicles, while in the tissue that was more chronically affected, atrophy was observed throughout the entire fascicle. The majority of ganglia examined [Meissner’s/submucosal and Auerbach’s/muscularis propria within the colon wall (Figure 5), myenteric plexuses within the esophagus, and the ganglia of the vagus nerve] were infiltrated by inflammatory cells (eosinophils, neutrophils, macrophages, and lymphocytes). No histopathologic abnormalities were observed within examined brain tissue. An interesting finding was the presence of wedge-shaped infarcts in the kidney with loss of tubules and replacement by fibrous connective tissue.

## Discussion

This case represents an unusual combination of clinical presentation, neurologic examination findings, and muscle histopathology. The dog was diagnosed with immune-mediated PM. However, in addition to skeletal myositis, inflammatory cells were also found infiltrating the myocardium, endocardium, and epicardium, as well as plexuses of the colon, esophagus, and vagus nerve.

Concurrent inflammation of both skeletal muscle and cardiac muscle is a rare finding in the veterinary literature. To the authors’ knowledge, only one previous case of myocarditis associated with PM has been reported in a dog (4). In this previous case report, at necropsy the dog was diagnosed with an enlarged right atrium. Mononuclear cell infiltration and fibrosis was noted in the endocardium and myocardium of all heart chambers. Prior to necropsy the dog was noted as having atrial premature complexes. In the current case, no chamber enlargement was noted; however, atrial premature complexes were observed premortem. Though not commonly reported in dogs, cardiac inflammation is well established in humans with PM (9). Human patients with PM and concurrent myocarditis have a more guarded prognosis with the major cause of death secondary to congestive heart failure, myocardial infarction, and arrhythmias (10). One prospective report revealed that 76% of human PM cases have cardiac abnormalities, most commonly, atrioventricular conduction disturbances including atrial and ventricular arrhythmias on electrocardiography (11).

In addition to cardiac involvement in our case, cellular infiltrations were present within plexuses of the colon and esophagus, and within the vagus nerve. There was no evidence of inflammation in the muscular layers of the intestines; the infiltrates were confined to the nerve plexuses. The clinical diarrhea, anorexia, and cardiac arrhythmia were likely sequela to abnormal neurologic innervation of the gastrointestinal musculature secondary to inflammation within the gastrointestinal plexuses and the vagal nerve.

The pattern of perifascicular atrophy was a prominent finding within skeletal muscles and suggestive of ischemia from small vessel disease. The wedge-shaped infarcts located in the kidneys are also suggestive of involvement of small perforating vessels in the underlying disease process. Perifascicular atrophy has been noted in several different types of inflammatory myopathies in humans (12), however, it is most commonly observed in dermatomyositis (13). Perifascicular atrophy is not a common feature of canine dermatomyositis (1). Skin lesions were not present in the dog of our report.

Two diseases in humans in which perifascicular atrophy and necrosis is observed include: antisynthetase syndrome and limb girdle muscular dystrophies (LGMD). The former is an uncommon disease of connective tissue. Affected humans produce antibodies to anti-aminoacyl t-RNA synthetase and manifest with signs of myositis, arthritis, and also interstitial lung disease (14, 15). Samples of musculature from humans with LGMD also can show evidence for perifasciular atrophy on histopathology. This pattern of atrophy and its predominance is indicative of both severity and also chronicity of the disease process (16) Using immunofluorescent stainings, upregulation of MHC I was noted which is supportive of an immune-mediated cause in this case. Upregulation of MHC I is used in human diagnostics to support the diagnosis of immune-mediated myositis even if cellular infiltrates are minimal or not evident in muscle biopsy sections (17).

## Concluding Remarks

This is a novel case of diffuse myositis, myocarditis, and epicarditis with a distinct perifascicular pattern of atrophy in skeletal muscle suggesting vascular ischemic disease. The upregulation of MHC-1 in immunofluorescent staining is consistent with an underlying immune etiology. Based on the findings in this case and the previously published case (4) confirming concurrent PM and myocarditis in dogs, and the incidence of cardiac involvement in human PM (11), clinicians are advised to evaluate the heart in cases of canine PM.

## Author Contributions

PR manuscript preparation and main clinician on case during its presentation. ST participation in manuscript preparation and neurologic consultation on clinical case. RS pathologic evaluation of necropsy samples and participation in manuscript preparation. LG performance of immunofluorescent studies and pathologic evaluation. GS pathologic evaluation of muscle and nerve biopsies, participation in manuscript preparation.

## Conflict of Interest Statement

The authors declare that the research was conducted in the absence of any commercial or financial relationships that could be construed as a potential conflict of interest.
